# Age and Sex as Moderators of Sleep Architecture in Schizophrenia, Bipolar Disorder, and Unipolar Depression: A Case-Control Polysomnographic Systematic Review and Meta-Regression

**DOI:** 10.3390/biology15171487

**Published:** 2026-09-02

**Authors:** Dario Morra, Giuseppe Barbato

**Affiliations:** Department of Psychology, Università degli Studi della Campania “Luigi Vanvitelli”, Viale Ellittico 31, 81100 Caserta, Italy

**Keywords:** sleep, polysomnography, schizophrenia, bipolar disorder, major depressive disorder, meta-regression

## Abstract

Sleep architecture alterations have been shown in major psychiatric disorders, but the contribution of demographic factors to between-study heterogeneity remains insufficiently characterized. This study reports a transdiagnostic systematic review and meta-regression of case-control polysomnographic studies comparing schizophrenia, bipolar disorder, and unipolar depression with healthy controls. The factors age and sex as possible moderators across harmonized case-control polysomnographic studies have been analyzed. Eligible studies reported diagnostic criteria, treatment status, age, and sex. Because the UD literature encompassed developmentally distinct populations, pediatric/adolescent and adult studies were analyzed separately. Standardized mean differences were synthesized using random-effects models with REML estimation. Univariable mixed-effects meta-regressions assessed study-level mean age and sex composition as moderators within each diagnostic group. Study-level mean age and sex composition significantly moderated delta sleep effect sizes in drug-naive schizophrenia, while study-level mean age significantly moderated REM time in drug-free schizophrenia. In bipolar disorder, study-level mean age moderated REM sleep time effect sizes during mania, whereas sex composition moderated delta sleep and REM density effect sizes. In UD, study-level mean age moderated total sleep time, and sex composition moderated wake after sleep onset only in pediatric/adolescent studies, whereas no significant moderation was observed in adult samples. No significant moderator effects were identified during bipolar depression. Overall, demographic composition explained only a modest proportion of between-study heterogeneity. Interpretation is limited by the ecological nature of study-level meta-regression and residual study heterogeneity.

## Simple Summary

Sleep disturbances are among the most consistent biological findings in schizophrenia, bipolar disorder, and unipolar depression. However, polysomnographic studies often report heterogeneous results, partly because of the imbalanced demographic composition of the populations investigated. We performed a transdiagnostic systematic review and meta-regression to investigate whether study-level age and sex contribute to variability in reported sleep abnormalities across these major psychiatric disorders. While several demographic associations were identified, their pattern differed across diagnoses and sleep parameters, suggesting that age and sex account for only a limited proportion of between-study heterogeneity. Separate analyses of pediatric/adolescent and adult unipolar depression further highlighted the importance of developmental stage when interpreting sleep abnormalities. These findings provide a framework for interpreting inconsistencies across the psychiatric sleep literature and underscore the importance of harmonized study designs and individual participant data meta-analyses for future research.

## 1. Introduction

Monoaminergic, cholinergic, and glutamatergic systems, as well as circadian and homeostatic mechanisms involving melatonin and cortisol, play fundamental roles in the sleep regulation mechanisms [1,2] and in the pathophysiology of major psychiatric disorders [3,4]. REM sleep is promoted by cholinergic activity and inhibited by serotonergic and noradrenergic activity [5,6]. Accordingly, shortened REM latency (REML), one of the most consistent polysomnographic findings in major depressive disorder (MDD), has been attributed to reduced monoaminergic neurotransmission [7,8]. Glutamatergic thalamocortical circuits contribute to the generation of sleep oscillations, including sleep spindles during NREM sleep [9], which are consistently altered in schizophrenia (SCZ) [10]. Early studies identified a modulating role of estrogens on dopamine activity [11,12,13], suggesting a reduced risk for SCZ in females [14]. Estrogen can increase noradrenaline in locus coeruleus, a target region for the expression of arousal, which may be increased in females [15,16]. Aging is associated with alterations in circadian and neuroendocrine regulation, including earlier timing of melatonin and cortisol secretion and reduced homeostatic and circadian sleep-promoting processes [17], which contribute to age-related changes in sleep architecture [18].

As shown by polysomnographic (PSG) studies, reductions in total sleep time (TST), sleep efficiency (SE), delta sleep percentage (%DELTA), REM sleep time (REMT), REM sleep percentage (%REM), and REML are seen with age, whereas sleep onset latency (SOL), wake after sleep onset (WASO) and the percentage of stage 1 (%ST1) and stage 2 (%ST2) sleep generally increase with age [19,20]. These findings are broadly supported by an earlier meta-analysis of PSG and actigraphy measures [21]; however, age-related changes in %ST2, %DELTA, and %REM remain debated [22].

Sex-related differences in sleep architecture have also been consistently reported. Compared with age-matched males, females generally exhibit deeper and more consolidated sleep, characterized by higher %DELTA and lower %ST1, %ST2, and wakefulness after sleep onset [19]. Male sex has additionally been associated with shorter REML [22]. Similarly, in a meta-analysis which included PSG and actigraphy data, longer TST, albeit with slightly prolonged SOL, was reported in females compared with age-matched men [21].

Large-scale normative data for REM density (REMD) remain scarce, and scoring methodologies are not always consistent across studies. Although some investigations reported an age-related reduction in REMD [23,24], others failed to replicate this finding [25,26]. Likewise, evidence on sex differences in REMD is limited, with one study reporting no significant differences between males and females [26]. More generally, healthy adults exhibit substantial intra- and inter-individual variability across PSG sleep measures [19].

Meta-analyses have consistently identified abnormalities in sleep architecture in several major psychiatric disorders, including SCZ [27,28], schizoaffective disorder (SZA) [29], MDD [30,31,32,33] and bipolar disorder (BD) [34,35]. However, the extent to which demographic characteristics contribute to between-study variability in these abnormalities has received comparatively little attention. A systematic review on demographic subgroup differences reported altered REM sleep measures in men with MDD compared to healthy controls (HC), whereas women with MDD more frequently showed impaired sleep continuity and prolonged stage 1 sleep. Among men with SCZ, prolonged REMT but normal REML were described. The same review found only limited evidence supporting age-related differences in sleep architecture among patients with MDD [36].

The present study aimed to investigate age and sex as study-level moderators of case-control differences in PSG sleep architecture across schizophrenia, bipolar disorder, and unipolar depression using harmonized mixed-effects meta-regression analyses.

## 2. Materials and Methods

Studies included in the present analysis were derived from previously published harmonized systematic reviews and meta-analyses conducted by the authors, investigating sleep architecture in schizophrenia (SCZ) and bipolar disorder (BD) [28,35]. These datasets were re-assembled and harmonized according to updated inclusion criteria. All eligible studies were re-screened to ensure consistency with current requirements for reporting of age, sex, and treatment status. Studies not meeting these criteria were excluded prior to analysis.

Previous meta-analyses on major depressive disorder (MDD) have applied heterogeneous inclusion criteria, including restriction to early-onset samples, focus on selected sleep parameters (e.g., REM sleep), or incomplete reporting of PSG outcomes [30,31,32,33]. In addition, several syntheses have combined heterogeneous depressive phenotypes, including seasonal affective disorder and broadly defined or non-operationalized MDD, resulting in substantial diagnostic variability across included samples.

To address these limitations, a harmonized dataset of recurrent, non-psychotic, drug-free unipolar depression (UD) case-control studies was additionally assembled, with the aim of improving diagnostic specificity and methodological consistency. This dataset provided a unified basis for subsequent meta-regression analyses examining age and sex as study-level moderators of sleep architecture. Inclusion criteria for UD were aligned with those applied in SCZ and BD datasets to ensure comparability across diagnostic groups.

Detailed characteristics of individual studies are provided in the Supplementary Materials (Supplementary File S1).

### 2.1. Database Search (UD vs. HC)

**Figure 1:**
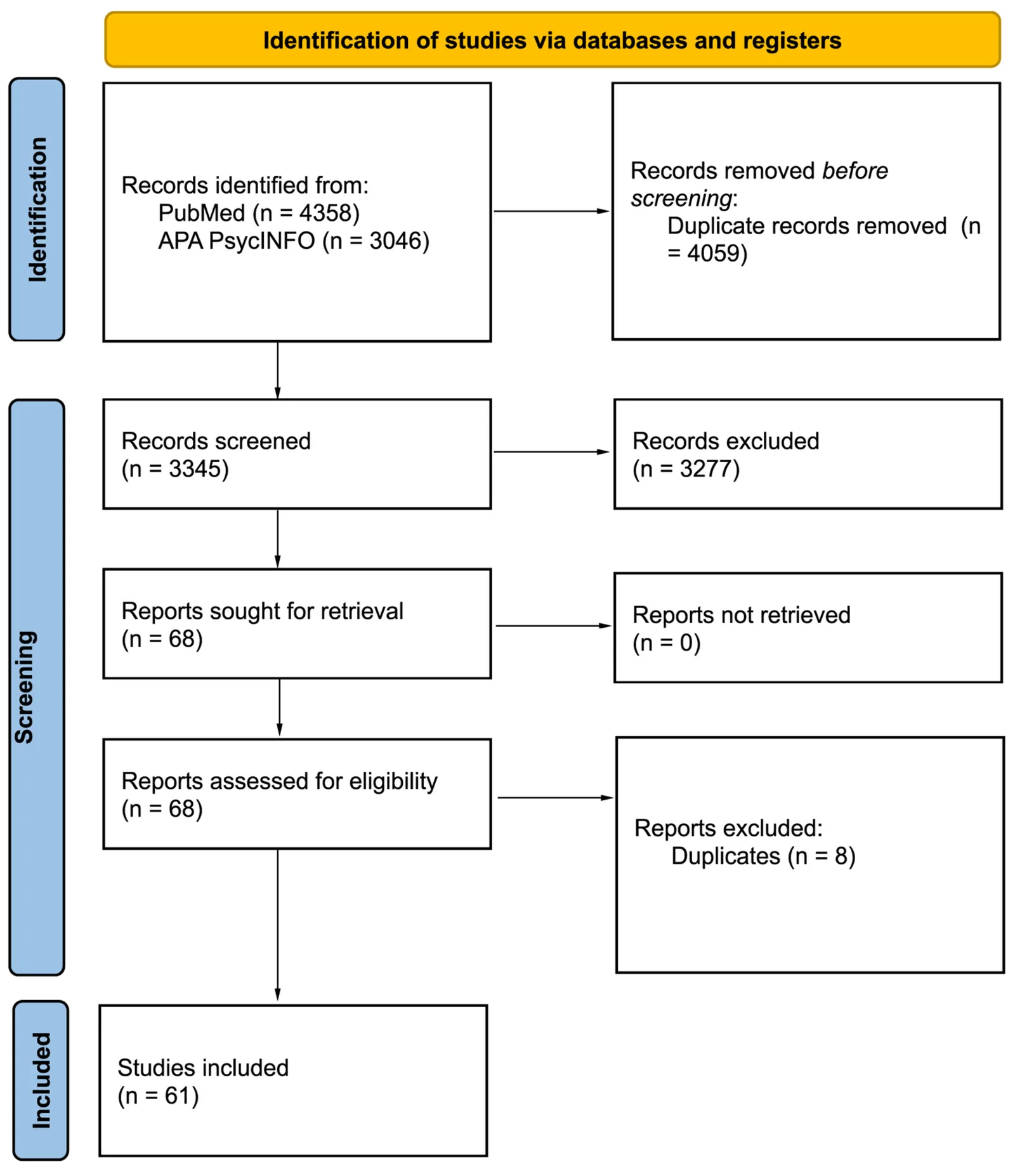
PRISMA diagram for the UD vs. HC case-control studies search.

A systematic literature search was conducted in PubMed and PsycINFO from 1 November 1946 to 15 June 2026. The same search strategy was applied to both databases using the terms “(sleep OR sleep*) AND (polysomnogra* OR EEG) AND depress*”. No language restrictions were applied. The search yielded 4358 records from PubMed and 3046 from PsycINFO. After removal of duplicates (4059), identified by matching titles, abstracts, and authors, 3345 records remained for screening. Full-text assessment identified 68 eligible studies. Of these, 3277 were excluded due to absence of PSG sleep data. Seven studies were excluded due to overlapping patient samples with previously published datasets. Ultimately, 61 studies were included in the UD vs. HC meta-analysis. Study eligibility was assessed independently by both authors (DM and GB). The PRISMA flow diagram is presented in Figure 1, and the PRISMA checklist is provided in Supplementary File S2. The PRISMA flow diagram (Figure 1) illustrates only the study selection process for the newly conducted systematic review of drug-free UD versus HC, which was performed to assemble the harmonized UD dataset for the present study. The studies included for SCZ and BD were derived from our previously published harmonized systematic reviews and meta-analyses [29,36]. As these datasets were not identified through a new literature search conducted as part of the present study, their original PRISMA flow diagrams are reported in the corresponding publications and are therefore not reproduced here. The review protocol was not prospectively registered.

### 2.2. Eligibility Criteria

To ensure methodological comparability across diagnostic groups, identical eligibility criteria were applied to all harmonized datasets (SCZ, BD, and UD):

1. Case-control studies including at least one patient sample composed exclusively of individuals with SCZ, UD, or BD.
2. Case-control studies including at least one healthy control (HC) sample.
3. Studies clearly reporting the diagnosis, age, sex, and treatment status of the patient sample.
4. Studies in which the patient sample was entirely drug-naive or drug-free.
5. Studies using PSG for sleep staging.
6. Studies in which patients were diagnosed according to DSM or iCD criteria or according to Research Diagnostic Criteria (RDC).

All included studies scored at least 6 points on the Newcastle–Ottawa Scale.

Studies were excluded from the meta-analysis if they met at least one of the following criteria:

1. Absence of a healthy control (HC) sample.
2. Patient samples not composed exclusively of individuals with SCZ, UD, or BD.
3. Failure to clearly report the diagnosis, age, sex, or treatment status of the patient sample.
4. Patient samples including treated participants or participants who were not entirely drug-naive or drug-free.
5. Studies including patients with other psychiatric, neurological, sleep-related, or substance use comorbidities (e.g., ADHD, obstructive sleep apnea, substance abuse, etc.).
6. Studies not using PSG for sleep staging (e.g., actigraphy, sleep diaries)

### 2.3. Data Extraction

Sample size, age, sex (as male proportion), diagnosis, and medication status were extracted from the studies assessed as eligible, together with the mean and standard deviation (SD) of the following polysomnographic parameters: TST, SE, SOL, WASO, ST1, %ST1, ST2, %ST2, DELTA, %DELTA, REMT, %REM, REML, REMD.

### 2.4. Statistical Analysis

Statistical analyses were performed using Jamovi 2.6.26 (Windows x64). For each PSG parameter and psychiatric diagnostic group, effect sizes were expressed as standardized mean differences (SMD) between patients and HC, as reported in the original studies. Random-effects models were fitted using restricted maximum-likelihood (REML) estimation. Univariable mixed-effects meta-regression models were then performed to examine whether study-level mean age and the proportion of male participants were associated with observed effect sizes. Age and sex were entered separately as continuous moderators based on sample-size weighted study-level aggregates. For meta-regression models including fewer than 10 studies, the Knapp–Hartung adjustment was applied to improve the accuracy of statistical inference. Specifically, study-level mean age and male proportion were calculated as sample-size weighted averages of the corresponding patient and healthy control groups according to the following formulas:

Overall Age = [(Agepatients × Npatients) + (Agecontrols × Ncontrols)]/(Npatients + Ncontrols)

Overall %Male = [(%Malepatients × Npatients) + (%Malecontrols × Ncontrols)]/(Npatients + Ncontrols)

Results are reported as regression coefficients with corresponding *p*-values. No multivariable models or interaction terms were tested.

The analytical strategy was based on harmonized datasets derived from previously published meta-analyses of sleep in SCZ and BD, with standardized inclusion criteria across diagnostic groups and restriction to drug-naive or drug-free samples. The primary objective was estimation of age and sex as study-level moderators of sleep architecture differences.

Between-study heterogeneity, publication bias, and influence diagnostics were not uniformly re-estimated in the transdiagnostic SCZ and BD analyses, in order to maintain consistency with the analytical structure of the original datasets. An exception was made for the UD subgroup, for which a fully harmonized meta-analysis was additionally conducted. In this subgroup, the primary outcome was a standard mean difference (SMD), and data were fitted with a random-effects model. Heterogeneity assessment, influence diagnostics, and publication bias analyses were performed, including funnel plot inspection, Begg and Mazumdar rank correlation test, and Egger-type regression of effect sizes on their standard errors.

Because the UD literature included both pediatric/adolescent and adult populations, pooling these studies could introduce developmental confounding due to age-related changes in sleep architecture. Therefore, all meta-analyses and meta-regressions for UD were performed separately in pediatric/adolescent (mean study age ≤ 19.1 years) and adult (mean study age ≥ 23.3 years) samples. These thresholds reflected the natural separation of the included literature, with no study having a mean age between 19.1 and 23.3 years.

Because the moderator analyses addressed distinct prespecified biological hypotheses across diagnostic groups and PSG parameters rather than repeated testing of a common outcome, correction for multiple comparisons was not incorporated into the primary inferential framework. Nevertheless, Benjamini–Hochberg false discovery rate (FDR) correction was additionally performed within each diagnostic group and moderator (age or sex). This conservative analysis was intended to evaluate the robustness of nominally significant findings.

Leave-one-out sensitivity analyses were additionally performed, where feasible, by sequentially omitting one study at a time to assess whether nominally significant moderator associations were driven by individual influential studies.

## 3. Results

A total of 132 studies were included after application of the updated inclusion criteria and were stratified according to diagnostic group [37,38,39,40,41,42,43,44,45,46,47,48,49,50,51,52,53,54,55,56,57,58,59,60,61,62,63,64,65,66,67,68,69,70,71,72,73,74,75,76,77,78,79,80,81,82,83,84,85,86,87,88,89,90,91,92,93,94,95,96,97,98,99,100,101,102,103,104,105,106,107,108,109,110,111,112,113,114,115,116,117,118,119,120,121,122,123,124,125,126,127,128,129,130,131,132,133,134,135,136,137,138,139,140,141,142,143], with some studies providing separate datasets for different diagnoses and therefore contributing to multiple meta-analyses. Study-level characteristics, including sample sizes for patients and HC, mean age, and proportion of male participants, are summarized in Table 1. For each study, overall mean age and male proportion were calculated by combining the patient and HC groups using sample-size weighting. Diagnosis-level mean age and male proportion reported in Table 1 were then calculated as sample-size weighted averages across studies, with each study weighted by its total sample size (patients plus HC). Results of univariable meta-regression analyses examining age and sex as moderators of each sleep parameter are reported in Table 2 and Table 3, respectively.

Of the 132 studies, 14 investigated drug-naive SCZ versus HC, 40 investigated drug-free SCZ versus HC, 5 investigated drug-free BD, manic episode, 12 investigated BD, depressive episode, and 61 investigated sleep in UD versus HC. To reduce potential developmental confounding, UD studies were analyzed separately as pediatric/adolescent (20 studies) and adult (41 studies) populations. The corresponding harmonized meta-analysis results are presented in Table 4 (pediatric/adolescent) and Table 5 (adult), while assessments of between-study heterogeneity, identification of potential outliers and influential studies, and evaluation of publication bias using funnel plot inspection, Begg and Mazumdar rank correlation test, and Egger-type regression of effect sizes on their standard errors are reported in Table 6 (pediatric/adolescent) and Table 7 (adult).

### 3.1. Age and Sex as Moderators

Both study-level mean age (*p* = 0.043) and sex composition (*p* = 0.026) significantly moderated %DELTA effect sizes in drug-naive SCZ, while study-level mean age (*p* = 0.036) composition significantly moderated REMT effect sizes in drug-free SCZ. In BD, no significant associations between study-level mean age or sex composition and sleep parameter effect sizes were observed during the depressive phase. During the manic phase, study-level mean age significantly moderated REMT effect sizes (*p* = 0.043), while sex composition significantly moderated DELTA (*p* = 0.018) and REMD (*p* = 0.017) effect sizes. In UD, study-level mean age significantly moderated TST effect sizes (*p* = 0.041) in the adolescent/pediatric subgroup and WASO effect sizes (*p* = 0.003) in the adult subgroup. No significant associations between study-level mean sex composition and sleep parameter effect sizes were observed in either UD subgroup.

### 3.2. False Discovery Rate Correction and Sensitivity Analysis

Following FDR correction, the associations between study-level mean age and %DELTA effect sizes in drug-naive SCZ (adjusted *p* = 0.602), REMT effect sizes in drug-free SCZ (adjusted *p* = 0.504), REMT effect sizes during the manic phase of BD (adjusted *p* = 0.524), and TST effect sizes in pediatric/adolescent UD (adjusted *p* = 0.455), as well as the associations between sex composition and %DELTA effect sizes in drug-naive SCZ (adjusted *p* = 0.364), and DELTA (adjusted *p* = 0.126) and REMD (adjusted *p* = 0.126) effect sizes during the manic phase of BD, were no longer statistically significant. Only the association between study-level mean age and WASO effect sizes in adult UD remained significant after FDR correction (adjusted *p* = 0.042).

Leave-one-out sensitivity analyses were performed for all diagnostic subgroup meta-regression models in which reliable model estimation remained feasible following sequential omission of individual studies. In some subgroup analyses, particularly those regarding BD mania, including only four studies, leave-one-out analysis could not be performed because exclusion of a single study resulted in an insufficient number of studies for stable mixed-effects meta-regression estimation. Across all evaluable subgroup analyses, sequential omission of individual studies did not materially alter the regression coefficients or the statistical significance of the nominally significant associations, indicating that the observed results were not driven by individual influential studies.

### 3.3. Drug-Free UD vs. HC Meta-Analysis

The meta-analysis of sleep parameters in drug-free pediatric/adolescent UD versus HC revealed significant sleep continuity alterations, with increased SOL (*p* < 0.001) and decreased SE (*p* = 0.003). ST2 was significantly reduced (*p* = 0.005), whereas %REM was significantly increased (*p* = 0.013).

The meta-analysis of sleep parameters in drug-free adult UD versus HC revealed significant sleep continuity alterations as well, with decreased TST (*p* < 0.001) and SE (*p* < 0.001), and increased SOL (*p* < 0.001) and WASO (*p* < 0.001). ST2 (*p* < 0.001), DELTA (*p* < 0.001), and %DELTA (*p* = 0.014) were significantly reduced. %REM (*p* < 0.001) and REMD (*p* < 0.001) were significantly increased, whereas REML was shortened (*p* < 0.001).

### 3.4. Age and Sex Imbalance Assessment

Across the included literature, demographic composition showed substantial and diagnosis-dependent heterogeneity. In SCZ, mean study-level age was similar between drug-naive (30.8 ± 9.4 years, range 22.2–70.9) and drug-free samples (29.9 ± 4.3 years, range 16.2–44.6), although age variability was markedly lower in drug-free studies, indicating a more restricted age distribution. Sex distribution was male-biased in both SCZ groups, with drug-free studies showing higher male representation (73.0 ± 21.7% male, median 100%) than drug-naive studies (68.3 ± 13.4% male). In BD, study-level mean age was higher during depressive episodes (38.4 ± 8.3 years) than during manic episodes (34.2 ± 6.5 years), while sex composition remained variable in both groups (52.1 ± 25.1% and 65.4 ± 21.0% male, respectively). In UD, both pediatric/adolescent (13.7 ± 2.7 years, range 9.4–19.1) and adult (38.2 ± 8.7 years, range 23.3–69.5) studies showed substantial variability in male proportion (46.1 ± 31.7%) and 59.0 ± 32.7%.

## 4. Discussion

To date, this is the first study to systematically examine age and sex as study-level moderators of PSG case-control differences across schizophrenia, bipolar disorder, and unipolar depression.

A study-level association between age and TST effect sizes in pediatric/adolescent UD was found, consistent with the physiological decline in TST accompanying normal maturation. In contrast, a study-level association between age and WASO effect sizes was observed in adult UD, probably reflecting age-related differences in the sleep disturbances associated with UD.

Cross-sectional reports on age and sex differences in SCZ and BD are scarce, while disproportionately investigated in MDD. No effect of age and sex on any sleep parameter in the depressive phase of BD was found. On the other hand, study-level mean age significantly moderated REMT effect sizes during manic episodes of BD. No other effect of age on any sleep parameter in mania was found, consistent with what was reported in an early study [88]. Previous studies investigating sex differences in sleep during mania have largely reported null findings [89], whereas our study identified sex composition as a significant study-level moderator of DELTA effect sizes in BD mania. Sex composition also significantly moderated REMD effect sizes. Sex-related differences in the clinical expression, course, and neurobiology of bipolar disorder have been reported [144,145] and may contribute to the observed study-level moderation of REMD. REMD has been proposed as a marker associated with antidepressant treatment response in MDD [146]. Whether REMD may similarly reflect treatment response in bipolar mania remains unknown. Given that lithium has been reported to delay REM sleep [147] and reduce REMD [148], future longitudinal studies should examine whether REMD changes during lithium treatment can be associated with clinical response.

Regarding SCZ, study-level mean age and sex composition significantly moderated %DELTA effect sizes in drug-naive samples, whereas no such effects were observed in drug-free samples. One possible explanation for this study-level difference is variation in illness stage and treatment history between the included populations. Drug-naive study samples are more likely to represent earlier phases of the disorder, whereas drug-free samples may include individuals with longer illness duration and prior pharmacological exposure or discontinuation effects, all of which may contribute to increased between-study heterogeneity in sleep architecture measures [27,28]. Such heterogeneity may attenuate detectable demographic moderation in chronic or clinically heterogeneous samples, raising the possibility that demographic influences may be more detectable in early-stage schizophrenia, although differences in study heterogeneity and statistical power may also contribute. Study-level mean age significantly moderated REMT effect sizes in drug-free SCZ, potentially reflecting greater variability in illness duration and treatment history in these samples.

The age- and sex-related differences observed in the present study may partly reflect physiological changes in the neurobiological systems regulating sleep. Notably, significant moderator effects were primarily identified for slow-wave sleep (%DELTA, DELTA) and REM-related measures (REMT, REMD), sleep states known to depend on partially distinct regulatory mechanisms. However, these effects were diagnosis-specific rather than uniformly distributed across psychiatric disorders, suggesting that demographic factors may interact with disorder-related neurobiological mechanisms instead of exerting generalized effects on sleep architecture.

Because the present study involved a large number of moderator analyses, Benjamini–Hochberg false discovery rate (FDR) correction was additionally performed within each diagnostic group and moderator. Following this analysis, only the association between study-level mean age and WASO effect sizes in adult UD remained statistically significant, whereas the remaining nominally significant moderator effects did not survive FDR correction. Accordingly, these remaining associations should be regarded as exploratory and interpreted as hypothesis-generating rather than confirmatory evidence.

### Limitations and Future Perspectives

Since the present analyses were based on aggregate study-level moderators, hypotheses on biological mechanisms potentially involved should be regarded as possible explanations for the between-study associations rather than evidence of individual-level interactions between age or sex and psychiatric diagnosis.

High-quality PSG studies in specific populations, particularly drug-naive SCZ and BD, remain limited in the literature. Several subgroup analyses were based on a limited number of available studies, particularly in BD, while drug-free SCZ studies showed marked male predominance. Therefore, regression coefficients for these subgroups should be interpreted cautiously, as limited statistical power and restricted variability in moderator distributions may increase uncertainty and risk of both Type I and Type II error. Furthermore, only univariable meta-regression models were performed. Consequently, the observed associations were not adjusted for other study-level covariates, and residual confounding between demographic characteristics cannot be excluded. Moreover, age and sex are not homogeneously represented across studies within and between diagnostic groups, with a general predominance of male participants and limited variability in demographic distributions. This demographic imbalance limits the ability to robustly assess differential effects of age and sex on PSG parameters. As with aggregate-data meta-regressions, the observed associations remain susceptible to ecological bias and may also reflect differences in the demographic composition of HC samples, patient-control demographic imbalance, or other study-level characteristics that cannot be disentangled using aggregate-data meta-regression. Finally, differences in observed moderation effects across disorders may partly reflect differences in between-study variance of demographic composition rather than disorder-specific mechanisms. In SCZ and BD, restricted variability in sex ratios across studies likely reduced statistical power to detect sex-related moderation effects, potentially attenuating regression estimates.

The meta-analysis of sleep parameters of drug-free UD versus HC confirmed disrupted sleep continuity and duration in adults, whereas adolescent/pediatric UD patients showed increased SOL and reduced SE only, suggesting initial insomnia. Adult patients also showed a marked increase in REM pressure and reduced SWS [32,36]. Although heterogeneity was moderate to high (>60%) for most analyzed parameters, low heterogeneity was observed in the meta-analysis of DELTA (13.20%) in UD patients, supporting consistent SWS reduction in depressed patients regardless of previous exposure to treatment. No significant difference in DELTA was observed between pediatric/adolescent UD and HC, and the corresponding meta-analysis showed no between-study heterogeneity (0%). ST2 was similarly reduced in both adolescent/pediatric and adult UD patients, suggesting that this alteration may occur across different developmental stages of the disorder. A meta-regression could not be performed for SZA due to insufficient number of available sleep studies and limited variability in study-level covariates, including age and sex distribution [29], highlighting a gap in the literature that currently limits characterization of sleep architecture in this diagnostic group.

Future research should prioritize longitudinal designs and individual participant data to better disentangle biological aging from illness chronicity and pharmacological exposure while reducing the limitations inherent to aggregate-data meta-regression. A better understanding of age- and sex-related differences in neurotransmitter, neuroendocrine, circadian, and homeostatic regulation may also help guide the development of more individualized pharmacological strategies tailored to the neurobiological profiles of different psychiatric disorders. A shift toward sleep microstructural characterization is also warranted, as sleep microstructure may be more sensitive than macrostructure to age-related differences [149,150]. Parameters such as REMD may benefit from this approach, given the heterogeneity in scoring methodologies. Methods aggregating healthy populations to model demographic effects on sleep spindles [151,152] could be extended to psychiatric cohorts to improve characterization of age and sex effects in pathological sleep. Such work should be coupled with improved sampling of underrepresented clinical strata, including drug-naive and drug-free populations with broader and more balanced demographic distributions, alongside stricter stratification of medication history. Finally, integrating circadian timing metrics with sleep microstructural features may improve characterization of dynamic vulnerability across mood and psychotic phases [153], while dimensional approaches across diagnostic boundaries may further enhance explanatory resolution beyond categorical classifications.

## 5. Conclusions

The present findings indicate that demographic composition contributes to, but does not fully explain, the heterogeneity of polysomnographic abnormalities reported across major psychiatric disorders. The inconsistent pattern of study-level age and sex associations across diagnoses and sleep parameters suggests that demographic factors alone are insufficient to account for disorder-specific alterations in sleep architecture. The separate evaluation of pediatric/adolescent and adult unipolar depression further demonstrates that developmental stage should be considered when synthesizing PSG evidence. Together, these findings support the use of more homogeneous study populations, harmonized methodologies, and individual participant data meta-analyses to better characterize demographic influences on sleep architecture and improve the interpretability of future psychiatric sleep research.

## Figures and Tables

**Table 1:** Included studies with numerosity of samples, age and sex composition.

	N. Datasets	N. Patients/HC	Average Age (Patients/HC)	Average %Males (Patients/HC)
**SCZ (drug-naive)**	14	263/232	30.81	68.29
**SCZ (drug-free)**	40	726/633	29.89	73.04
**BD (drug-free, manic)**	5	62/67	34.23	65.41
**BD (drug-free, depressed)**	12	138/178	38.39	52.06
**MDD (drug-free) (<19.1)**	20	533/439	13.73	46.30
**MDD (drug-free) (>23.3)**	41	1290/904	38.16	52.79

**Table 2:** Results of age as sleep parameter moderator.

	Age as Moderator											
	SCZ (Drug-Naive)		SCZ (Drug-Free)		BD (Depressed, Drug-Free)		BD (Manic, Drug-Free)		MDD (Drug-Free) (<19.1)		MDD (Drug-Free) (>23.3)	
	Estimate	*p*-Value	Estimate	*p*-Value	Estimate	*p*-Value	Estimate	*p*-Value	Estimate	*p*-Value	Estimate	*p*-Value
**TST**	0.008 (−0.038 to 0.054)	0.721	0.058 (−0.072 to 0.187)	0.385	−0.04 (−0.132 to 0.052)	0.344	0.025 (−0.060 to 0.110)	0.337	−0.165 (−0.324 to −0.006)	**0.041**	−0.001 (−0.036 to 0.007)	0.191
**SOL**	0.019 (−0.013 to 0.052)	0.240	−0.039 (−0.099 to 0.020)	0.192	0.075 (−0.053 to 0.202)	0.203	0.034 (−0.317 to 0.385)	0.780	−0.04 (−0.125 to 0.045)	0.355	0.016 (−0.004 to 0.036)	0.109
**SE**	−0.080 (−0.271 to 0.107)	0.394	0.015 (−0.060 to 0.090)	0.702	0.045 (−0.407 to 0.497)	0.810	−0.022 (−0.348 to 0.305)	0.848	−0.078 (−0.160 to 0.005)	0.065	−0.027 (−0.057 to 0.002)	0.065
**WASO**	−0.013 (−0.041 to 0.015)	0.358	0.049 (−0.018 to 0.115)	0.154	−0.002 (−0.085 to 0.082)	0.965	−0.035 (−0.133 to 0.063)	0.265	0.067 (−0.023 to 0.156)	0.148	0.023 (0.008 to 0.037)	**0.003**
**ST1**	−0.024 (−0.076 to 0.029)	0.380	0.026 (−0.058 to 0.111)	0.541	−0.022 (−0.124 to 0.079)	0.535	−0.058 (−0.181 to 0.065)	0.181	0.001 (−0.118 to 0.121)	0.981	0.005 (−0.019 to 0.030)	0.668
**%ST1**	−0.014 (−0.033 to 0.005)	0.160	0.011 (−0.056 to 0.077)	0.758	−0.032 (−0.166 to 0.102)	0.505	−0.033 (−0.222 to 0.156)	0.618	0.030 (−0.017 to 0.078)	0.211	0.012 (−0.011 to 0.035)	0.302
**ST2**	0.007 (−0.044 to 0.057)	0.793	−0.011 (−0.078 to 0.056)	0.743	−0.031 (−0.180 to 0.118)	0.552	0.014 (−0.071 to 0.098)	0.557	−0.023 (−0.083 to 0.037)	0.453	−0.019 (−0.040 to 0.003)	0.093
**%ST2**	0.000 (−0.048 to 0.048)	0.989	−0.014 (−0.065 to 0.037)	0.597	−0.025 (−0.136 to 0.087)	0.530	0.050 (−0.203 to 0.304)	0.572	−0.006 (−0.108 to 0.097)	0.916	−0.006 (−0.035 to 0.024)	0.715
**DELTA**	0.012 (−0.109 to 0.134)	0.841	0.002 (−0.065 to 0.068)	0.964	−0.004 (−0.043 to 0.035)	0.814	−0.032 (−0.131 to 0.068)	0.304	0.020 (−0.029 to 0.069)	0.421	−0.018 (−0.040 to 0.005)	0.125
**%DELTA**	0.113 (0.003 to 0.222)	**0.043**	0.000 (−0.012 to 0.010)	0.862	−0.006 (−0.047 to 0.034)	0.727	−0.041 (−0.129 to 0.047)	0.237	0.045 (−0.038 to 0.129)	0.287	−0.012 (−0.034 to 0.010)	0.275
**REMT**	0.008 (−0.041 to 0.056)	0.753	0.068 (0.005 to 0.131)	**0.036**	−0.011 (−0.073 to 0.051)	0.737	−0.024 (−0.046 to −0.002)	**0.043**	−0.165 (−0.380 to 0.050)	0.133	−0.007 (−0.23 to 0.009)	0.405
**%REM**	0.002 (−0.070 to 0.073)	0.964	0.049 (−0.011 to 0.110)	0.108	0.010 (−0.035 to 0.055)	0.657	−0.035 (−0.105 to 0.035)	0.208	−0.068 (−0.278 to 0.142)	0.526	0.012 (−0.010 to 0.034)	0.287
**REML**	−0.019 (−0.081 to 0.043)	0.547	0.034 (−0.019 to 0.087)	0.205	−0.046 (−0.177 to 0.085)	0.489	0.059 (−0.089 to 0.207)	0.294	0.108 (−0.045 to 0.261)	0.165	−0.005 (−0.025 to 0.014)	0.588
**REMD**	−0.034 (−0.110 to 0.043)	0.385	−0.004 (−0.048 to 0.040)	0.869	0.042 (−0.035 to 0.119)	0.282	−0.070 (−0.195 to 0.055)	0.137	0.112 (−0.242 to 0.467)	0.534	−0.005 (−0.036 to 0.026)	0.753
**Statistically significant *p*-values in bold**												

**Table 3:** Results of sex as sleep parameter moderator.

	Sex as Moderator											
	SCZ (Drug-Naive)		SCZ (Drug-Free)		BD (Depressed, Drug-Free)		BD (Manic, Drug-Free)		MDD (Drug-Free) (<19.1)		MDD (Drug-Free) (>23.3)	
	Estimate	*p*-Value	Estimate	*p*-Value	Estimate	*p*-Value	Estimate	*p*-Value	Estimate	*p*-Value	Estimate	*p*-Value
**TST**	0.031 (−0.003 to 0.065)	0.074	0.005 (−0.010 to 0.019)	0.522	0.000 (−0.033 to 0.034)	0.973	−0.009 (−0.010 to 0.028)	0.183	0.012 (−0.001 to 0.024)	0.069	0.000 (−0.005 to 0.006)	0.838
**SOL**	−0.010 (−0.036 to 0.016)	0.448	−0.010 (−0.022 to 0.002)	0.102	0.003 (−0.052 to 0.057)	0.914	−0.007 (−0.118 to 0.105)	0.864	−0.002 (−0.010 to 0.006)	0.680	0.000 (−0.007 to 0.006)	0.854
**SE**	0.037 (−0.020 to 0.095)	0.206	0.005 (−0.010 to 0.019)	0.522	0.012 (−0.086 to 0.110)	0.759	0.009 (−0.093 to 0.111)	0.798	0.005 (−0.002 to 0.012)	0.133	0.004 (−0.006 to 0.013)	0.424
**WASO**	−0.005 (−0.033 to 0.024)	0.732	0.000 (−0.011 to 0.010)	0.931	0.010 (−0.020 to 0.039)	0.477	−0.013 (−0.029 to 0.003)	0.074	−0.002 (−0.011 to 0.007)	0.658	0.004 (−0.001 to 0.009)	0.141
**ST1**	0.010 (−0.036 to 0.055)	0.679	−0.007 (−0.021 to 0.007)	0.332	0.010 (−0.025 to 0.044)	0.446	−0.011 (−0.066 to 0.043)	0.469	−0.004 (−0.014 to 0.007)	0.512	0.002 (−0.005 to 0.008)	0.620
**%ST1**	0.000 (−0.015 to 0.013)	0.890	0.000 (−0.014 to 0.014)	0.991	0.008 (−0.041 to 0.058)	0.623	−0.012 (−0.070 to 0.046)	0.564	−0.004 (−0.009 to 0.002)	0.187	0.003 (−0.003 to 0.009)	0.312
**ST2**	0.014 (−0.028 to 0.055)	0.525	−0.001 (−0.013 to 0.010)	0.800	−0.020 (−0.064 to 0.023)	0.233	−0.007 (−0.011 to 0.025)	0.234	0.005 (−0.002 to 0.011)	0.151	0.000 (−0.006 to 0.006)	0.918
**%ST2**	−0.014 (−0.053 to 0.026)	0.500	−0.002 (−0.012 to 0.008)	0.687	−0.016 (−0.047 to 0.015)	0.200	0.01 (−0.073 to 0.093)	0.726	0.002 (−0.007 to 0.012)	0.644	−0.001 (−0.010 to 0.006)	0.668
**DELTA**	0.081 (−0.009 to 0.172)	0.079	−0.019 (−0.229 to 0.192)	0.863	−0.005 (−0.017 to 0.007)	0.370	−0.014 (−0.023 to −0.006)	**0.018**	0.001 (−0.007 to 0.005)	0.777	−0.004 (−0.009 to 0.001)	0.107
**%DELTA**	0.096 (0.011 to 0.181)	**0.026**	−0.017 (−0.058 to 0.024)	0.424	−0.003 (−0.016 to 0.011)	0.642	−0.011 (−0.039 to 0.018)	0.318	−0.003 (−0.013 to 0.006)	0.491	−0.004 (−0.011 to 0.002)	0.147
**REMT**	0.028 (−0.010 to 0.065)	0.150	0.006 (−0.007 to 0.020)	0.363	−0.002 (−0.028 to 0.024)	0.856	−0.006 (−0.018 to 0.006)	0.162	0.011 (−0.015 to 0.037)	0.391	−0.002 (−0.007 to 0.002)	0.324
**%REM**	0.034 (−0.002 to 0.090)	0.238	0.011 (−0.002 to 0.025)	0.106	−0.004 (−0.023 to 0.014)	0.610	−0.014 (−0.030 to 0.002)	0.072	−0.005 (−0.020 to 0.010)	0.509	0.002 (−0.005 to 0.009)	0.541
**REML**	0.028 (−0.021 to 0.077)	0.268	0.008 (−0.004 to 0.021)	0.188	0.019 (−0.029 to 0.066)	0.405	0.024 (−0.013 to 0.060)	0.136	−0.003 (−0.016 to 0.010)	0.664	−0.002 (−0.008 to 0.004)	0.576
**REMD**	0.004 (−0.021 to 0.029)	0.760	0.009 (−0.005 to 0.022)	0.216	0.004 (−0.033 to 0.041)	0.799	−0.033 (−0.060 to −0.006)	**0.017**	−0.024 (−0.054 to 0.007)	0.125	0.001 (−0.009 to 0.011)	0.843
**Statistically significant *p*-values in bold**												

**Table 4:** Sleep parameters in UD (pediatric/adolescent) versus HC meta-analysis results.

	Meta-Analysis				
	N. of Studies	N. of Subjects	Mean ± SD	RE Model	*p*-Value
**TST**	19	523 vs. 429	482.61 ± 42.55 vs. 480.09 ± 32.08	0.000 (−0.413 to 0.415)	0.997
**SOL**	20	533 vs. 439	26.39 ± 17.80 vs. 18.68 ± 13.33	0.519 (0.302 to 0.736)	**<0.001**
**SE**	16	324 vs. 234	93.41 ± 4.54 vs. 94.24 ± 3.59	−0.331 (−0.547 to −0.114)	**0.003**
**WASO**	18	485 vs. 388	18.00 ± 16.44 vs. 19.62 ± 18.29	0.005 (−0.255 to 0.266)	0.968
**ST1**	19	523 vs. 429	28.85 ± 17.66 vs. 28.34 ± 15.02	0.181 (−0.114 to 0.477)	0.229
**%ST1**	20	533 vs. 439	6.16 ± 4.45 vs. 6.03 ± 4.36	0.009 (−0.126 to 0.145)	0.891
**ST2**	19	523 vs. 429	240.11 ± 44.26 vs. 246.42 ± 41.45	−0.241 (−0.408 to −0.073)	**0.005**
**%ST2**	20	533 vs. 439	49.77 ± 8.73 vs. 51.45 ± 7.18	−0.210 (−0.474 to 0.055)	0.120
**DELTA**	14	452 vs. 393	105.50 ± 31.55 vs. 99.49 ± 32.50	0.034 (−0.140 to 0.172)	0.630
**%DELTA**	17	429 vs. 340	22.51 ± 7.47 vs. 21.28 ± 6.95	0.075 (−0.127 to 0.276)	0.467
**REMT**	13	436 vs. 377	101.50 ± 24.72 vs. 97.49 ± 19.65	0.513 (−0.141 to 1.167	0.124
**%REM**	10	158 vs. 102	19.43 ± 5.88 vs. 18.05 ± 4.30	0.504 (0.104 to 0.904)	**0.013**
**REML**	19	506 vs. 428	104.36 ± 42.96 vs. 108.19 ± 38.14	−0.310 (−0.687 to 0.067)	0.107
**REMD**	15	413 vs. 348	2.43 ± 0.63 vs. 2.42 ± 0.75	0.250 (−0.568 to 1.067)	0.549
**Statistically significant *p*-values in bold**					

**Table 5:** Sleep parameters in UD (adult) versus HC meta-analysis results.

	Meta-Analysis				
	N. of Studies	N. of Subjects	Mean ± SD	RE Model	*p*-Value
**TST**	36	1091 vs. 756	374. 21 ± 59.87 vs. 405.50 ± 40.21	−0.546 (−0.737 to −0.355)	**<0.001**
**SOL**	39	1201 vs. 843	26.76 ± 23.84 vs. 16.01 ± 12.16	0.557 (0.370 to 0.743)	**<0.001**
**SE**	33	1144 vs. 764	84.16 ± 10.05 vs. 90.32 ± 6.30	−0.761 (1–0.43 to −0.479)	**<0.001**
**WASO**	30	1003 vs. 623	43.71 ± 63.20 vs. 27.87 ± 23.32	0.580 (−0.429 to 0.730)	**<0.001**
**ST1**	29	1002 vs. 623	30.18 ± 17.66 vs. 34.16 ± 15.02	0.006 (−0.214 to 0.226)	0.955
**%ST1**	31	1059 vs. 702	8.04 ± 4.45 vs. 8.19 ± 4.36	0.171 (−0.033 to 0.375)	0.100
**ST2**	30	1012 vs. 633	210.11 ± 44.26 vs. 229.83 ± 41.45	−0.605 (−0.806 to −0.405)	**<0.001**
**%ST2**	32	1069 vs. 712	56.12 ± 8.73 vs. 57.01 ± 7.18	−0.184 (−0.447 to 0.079)	0.170
**DELTA**	17	520 vs. 396	32.30 ± 31.55 vs. 46.41 ± 32.50	−0.313 (−0.464 to −0.162)	**<0.001**
**%DELTA**	35	1159 vs. 762	10.18 ± 7.47 vs. 11.49 ± 6.95	−0.244 (−0.437 to 0.050)	**0.014**
**REMT**	19	731 vs. 399	89.25 ± 27.00 vs. 86.88 ± 22.11	−0.002 (−0.154 to 0.149)	0.977
**%REM**	34	1075 vs. 697	23.54 ± 5.88 vs. 21.04 ± 4.30	0.337 (0.141 to 0.534)	**<0.001**
**REML**	39	1270 vs. 884	63.58 ± 31.01 vs. 79.49 ± 32.45	−0.521 (−0.694 to −0.348)	**<0.001**
**REMD**	28	1046 vs. 614	6.22 ± 1.71 vs. 5.78 ± 1.73	0.754 (0.470 to 1.037)	**<0.001**
**Statistically significant *p*-values in bold**					

**Table 6:** Heterogeneity, publication bias, outlier and overly influential studies, and funnel plot asymmetry analyses (pediatric/adolescent UD vs. HC).

	Heterogeneity			Publication Bias				Funnel Plot Asymmetry	
	Q	i^2^	*p*-Value	Fail-Safe N	*p*-Value	Outlier	Overly Influential	Rank Correlation (*p*-Value)	Regression Test (*p*-Value)
**TST**	75.35	87.95%	<0.001	0	0.235	Arana-Lechuga et al. (2008) [135]	Arana-Lechuga et al. (2008) [135]	0.489	0.036 *
**SOL**	44.68	55.08%	<0.001	343	<0.001	Arana-Lechuga et al. (2008) [135]	Dahl et al. (1991) [115] Arana-Lechuga et al. (2008) [135]	0.233	0.058
**SE**	20.07	32.65%	0.169	72	<0.001	-	-	0.139	0.123
**WASO**	45.05	65.20%	<0.001	0	0.413	Emslie et al. (1987) [108]	Emslie et al. (1987) [108] Emslie et al. (1990) [112]	0.369	0.303
**ST1**	56.58	75.83%	<0.001	4	0.038	Arana-Lechuga et al. (2008) [135]	Delvenne et al. (1992) [119] Arana-Lechuga et al. (2008) [135]	0.034 *	<0.0001 *
**%ST1**	23.46	4.28%	0.218	0	0.157	-	-	0.020 *	0.015 *
**ST2**	31.71	27.45%	0.024	81	<0.001	Arana-Lechuga et al. (2008) [135]	Arana-Lechuga et al. (2008) [135]	0.447	0.006 *
**%ST2**	53.38	70.41%	<0.001	35	0.003	-	-	0.386	0.542
**DELTA**	13.82	0.00%	0.386	0	0.350	-	Goetz et al. (1987) [109] McCracken et al. (1997) [125]	0.830	0.808
**%DELTA**	26.03	33.69%	0.054	0	0.108	-	-	0.903	0.178
**REMT**	68.58	94.63%	<0.001	77	<0.001	Arana-Lechuga et al. (2008) [135]	Arana-Lechuga et al. (2008) [135]	0.100	0.0001 *
**%REM**	18.71	50.90%	0.028	36	<0.001	-	-	0.728	0.346
**REML**	71.74	85.02%	<0.001	55	<0.001	Arana-Lechuga et al. (2008) [135]	Arana-Lechuga et al. (2008) [135]	0.164	0.007 *
**REMD**	132.23	95.95%	<0.001	6	0.029	McCracken et al. (1997) [125]	McCracken et al. (1997) [125] Armitage et al. (2000) [126]	0.240	0.425

* Asymmetry found.

**Table 7:** Heterogeneity, publication bias, outlier and overly influential studies, and funnel plot asymmetry analyses (adult UD vs. HC).

	Heterogeneity			Publication Bias				Funnel Plot Asymmetry	
	Q	i^2^	*p*-Value	Fail-Safe N	*p*-Value	Outlier	Overly Influential	Rank Correlation (*p*-Value)	Regression Test (*p*-Value)
**TST**	100.51	68.62%	<0.001	1328	<0.001	Landolt & Gillin (2005) [131]	Landolt & Gillin (2005) [131]	0.818	0.629
**SOL**	106.90	70.47%	<0.001	1591	<0.001	Asaad et al. (2016) [91]	Asaad et al. (2016) [91]	0.792	0.214
**SE**	140.58	85.84%	<0.001	2171	<0.001	Asaad et al. (2016) [91]	Liscombe et al. (2002) [127] Asaad et al. (2016) [91]	0.489	0.046 *
**WASO**	50.10	41.38%	0.009	1065	<0.001	-	-	0.646	0.795
**ST1**	101.59	73.61%	<0.001	0	0.320	Kwon et al. (2019) [142]	Kwon et al. (2019) [142]	0.161	0.451
**%ST1**	112.67	71.99%	<0.001	98	<0.001	-	Hubain et al. (2006) [133] Asaad et al. (2016) [91]	0.458	0.396
**ST2**	87.70	67.26%	<0.001	1185	<0.001	-	-	0.671	0.719
**%ST2**	136.71	83.41%	<0.001	114	<0.001	Asaad et al. (2016) [91]	Asaad et al. (2016) [91]	0.489	0.956
**DELTA**	16.25	13.20%	0.436	99	<0.001	-	-	0.440	0.962
**%DELTA**	97.97	71.48%	<0.001	221	<0.001	Asaad et al. (2016) [91]	Asaad et al. (2016) [91]	0.446	0.189
**REMT**	18.71	18.38%	0.410	0	0.498	-	Hubain et al. (2006) [133]	0.836	0.705
**%REM**	92.18	69.38%	<0.001	458	<0.001	Landolt & Gillin (2005) [131]	Landolt & Gillin (2005) [131]	0.131	0.026 *
**REML**	107.27	67.60%	<0.001	1513	<0.001	Landolt & Gillin (2005) [131]	Landolt & Gillin (2005) [131]	0.648	0.517
**REMD**	116.37	83.45%	<0.001	1501	<0.001	Asaad et al. (2016) [91]	Lauer et al. (1995) [121] Asaad et al. (2016) [91]	0.246	0.057

* Asymmetry found.

## Data Availability

No new data were created or analyzed in this study. Data sharing is not applicable to this article.

## Supplementary Materials

The following supporting information can be downloaded at: https://www.mdpi.com/article/10.3390/biology15171487/s1, Supplementary File S1 (Included studies), Supplementary File S2 (PRISMA checklist). References [37,38,39,40,41,42,43,44,45,46,47,48,49,50,51,52,53,54,55,56,57,58,59,60,61,62,63,64,65,66,67,68,69,70,71,72,73,74,75,76,77,78,79,80,81,82,83,84,85,86,87,88,89,90,91,92,93,94,95,96,97,98,99,100,101,102,103,104,105,106,107,108,109,110,111,112,113,114,115,116,117,118,119,120,121,122,123,124,125,126,127,128,129,130,131,132,133,134,135,136,137,138,139,140,141,142,143,154] are cited in the supplementary materials.

## Abbreviations

The following abbreviations are used in this manuscript:

Abbreviation	In full
BD	Bipolar disorder
DELTA	Delta sleep time
%DELTA	Delta sleep percentage
HC	Healthy control
MDD	Major depressive disorder
NREM	Non-rapid-eye-movement
PRISMA	Preferred reporting items for systematic reviews and meta-analyses
PSG	Polysomnography
REM	Rapid-eye-movement
REMD	Rapid-eye-movement density
REML	Rapid-eye-movement latency
REMT	Rapid-eye-movement time
%REM	Rapid-eye-movement percentage
SCZ	Schizophrenia
SD	Standard deviation
SE	Sleep efficiency
SOL	Sleep onset latency
ST1	Stage 1 sleep time
ST2	Stage 2 sleep time
%ST1	Stage 1 sleep percentage
%ST2	Stage 2 sleep percentage
SWS	Slow-wave sleep
SZA	Schizoaffective disorder
TST	Total sleep time
UD	Unipolar depression
WASO	Wake after sleep onset
