# Supplementary material for: Age and Sex as Moderators of Sleep Architecture in Schizophrenia, Bipolar Disorder, and Unipolar Depression: A Case-Control Polysomnographic Systematic Review and Meta-Regression
Source: Biology (Basel). 2026 Sep 2;15(17):1487. doi: 10.3390/biology15171487 (PMC13565723; doi:10.3390/biology15171487)
Supplement: Supplementary file 1 [file biology-15-01487-s001.zip › Supplementary File S1.pdf]

SCZ (drug-naive)				
Study	N. SCZ	N. HC	Age (SCZ/HC)	%Males (SCZ/HC)
Vincent et al., 1968	15	11	24.60	80
Jus et al., 1968	42	10	26.15	74
Jus et al., 1973	11	10	70.95	100
Ganguli et al., 1987	8	16	22.15	56
Tandon et al., 1992	20	15	26.20	63.5
Lauer et al., 1997	22	20	31.70	64.5
Lauer et al., 1998	14	14	31.35	78
Poulin et al., 2001	7	10	34.55	68.5
Poulin et al., 2003	11	11	27.45	63.5
Forest et al., 2007	8	8	26.20	75
Yetkin et al., 2011	6	13	24.65	100
Manoach et al., 2014	15	25	27.00	68.5
Sasidharan et al., 2017	24	39	27.55	71.5
Ray et al., 2023	60	30	35.65	49

SCZ (drug-free)				
Study	N. SCZ	N. HC	Age (SCZ/HC)	%Males (SCZ/HC)
Feinberg et al., 1964-1965	14	5	27.90	74.00
Feinberg et al., 1964-1965	4	5	28.85	85.00
Azumi et al., 1967	3	4	36.50	100.00
Gulevich et al., 1967	13	7	36.50	100.00
Caldwell et al., 1967	25	10	28.55	100.00
Stern et al., 1969	8	6	23.20	46.00
Gaillard et al., 1984	8	16	27.50	50.00
Benson et al., 1985	11	13	28.35	100.00
Benson et al., 1985	8	19	28.40	100.00
Hiatt et al., 1985	5	18	26.50	100.00
Zarcone et al., 1987	12	18	32.00	100.00
Kempenaers et al., 1988	9	9	25.35	100.00
Riemann et al., 1991	11	11	33.70	73.00
Van Cauter et al., 1991	9	9	29.00	100.00
Benson et al., 1991	20	15	30.95	100.00
Tandon et al., 1992	20	15	27.70	68.50
Röschke & Aldenhoff, 1993	11	11	29.00	82.00
Benson et al., 1993	18	12	28.35	100.00
Riemann et al., 1994	43	36	35.55	56.00
Riemann et al., 1995	10	10	16.20	55.00
Röschke et al., 1995	13	13	28.00	85.00
Zarcone et al., 1995	18	10	32.70	100.00
Benson et al., 1996	14	15	32.05	100.00
Frieboes et al., 1997-1999	5	10	27.80	70.00
Nishino et al., 1998	14	14	33.85	100.00
Keshavan et al., 1998	30	30	30.90	57.00
Tandon et al., 1999	24	20	27.50	63.50
Rotenberg et al., 2000	20	10	44.55	62.50
Hoffmann et al., 2000	13	13	32.40	100.00
Lee et al., 2001	5	5	32.70	100.00
Salin-Pascual et al., 2004	18	21	32.00	46.00
Tekell et al., 2005	17	17	32.90	82.00
Das et al., 2005	15	15	31.55	53.00
Chen et al., 2006	25	44	28.50	52.00
Yang & Winkelman, 2006	15	15	40.40	100.00
Sarkar et al., 2010	20	20	27.90	100.00
Yetkin et al., 2011	7	13	25.05	100.00
Künzel et al., 2018	9	9	31.80	67.00
Sun et al., 2023	90	45	27.00	50.00
Sun et al., 2023	92	45	26.40	51.50

BD (drug-free, manic)				
Study	N. BD	N. HC	Age (BD/HC)	%Males (BD/HC)
Linkowski et al. (1986)	6	3	47.5	100
Hudson et al. (1988)	9	18	30.25	44
Hudson et al. (1992)	19	12	25.65	50
Linkowski et al. (1994)	8	14	40	100
Asaad et al. (2016)	20	20	37.4	65

BD (drug-free, depressed)				
Study	N. BD	N. HC	Age (BD/HC)	%Males (BD/HC)
Duncan et al. (1979)	22	36	44	38
Feinberg et al. (1982)	15	41	41.46	36
Linkowski et al. (1985)	10	3	46	100
Mendlewicz et al. (1985)	8	8	46	100
Linkowski et al. (1986)	6	3	46.5	100
Jernajczyk (1986)	10	10	40.25	50
De Maertelaer et al. (1987)	11	9	38	100
Linkowski et al. (1987)	5	3	47.3	100
Thase et al. (1989)	26	26	37.2	35
Lauer et al. (1992)	10	12	36.55	46
Hudson et al. (1993)	10	7	26	50
Rao et al. (2002)	5	20	15.2	38

	N. datasets	N. Patients/HC	Average Age (Patients/HC)	Average %Males (Patients/HC)
**SCZ (drug-naive)**	14	263/232	30.81	68.29
**SCZ (drug-free)**	40	726/633	29.89	73.04
**BD (drug-free, manic)**	5	62/67	34.23	65.41
**BD (drug-free, depressed)**	12	138/178	38.39	52.06
**MDD (drug-free) (<19.1)**	20	533/439	13.73	46.30
**MDD (drug-free) (>23.3)**	41	1290/904	38.16	52.79

MDD (drug-free) (<19.1)				
Study	N. MDD	N. HC	Age (MDD/HC)	%Males (MDD/HC)
Puig-Antich et al., 1982	27	11	9.4	63
Emslie et al., 1987	17	27	12.4	65
Goetz et al., 1987	25	40	15.4	55
Emslie et al., 1990	25	20	11.5	69
Dahl et al., 1990	27	30	15.2	42
Dahl et al., 1991	36	18	10.3	38
Goetz et al., 1991	38	37	19.1	44
Delvenne et al., 1992	11	11	18	0
Riemann et al., 1995	10	10	16.6	25
McCracken et al., 1997	20	13	15.8	39
Armitage et al., 2000	14	3	12.2	100
Armitage et al., 2000	11	2	12.2	0
Armitage et al., 2000	12	2	14.3	100
Armitage et al., 2000	13	8	14.3	0
Rao & Poland, 2008	16	16	15.7	41
Arana-Lechuga et al., 2008	16	7	11	43.7
Forbes et al., 2008	128	101	11.5	46
Lopez et al., 2010	21	21	12	52
Baena et al., 2024	40	34	16	0
Baena et al., 2024	26	28	16.2	100

MDD (drug-free) (>23.3)				
Study	N. MDD	N. HC	Age (MDD/HC)	%Males (MDD/HC)
Gillin et al., 1979	74	41	43.5	37
Feinberg et al., 1982	18	41	48	39.8
Borbely et al., 1984	9	9	31.7	33
Quitkin et al., 1985	19	21	37.6	42.5
Benson et al.,1985	10	13	34.3	100
Benson et al.,1985	7	19	32.2	100
Reynolds et al., 1985	25	25	69.5	24
Zarcone et al., 1987	12	18	34.7	100
De Maertelaer et al., 1987	8	9	38.5	100
Kupfer et al., 1989	8	8	24.4	56.5
Waller et al., 1989	60	20	30.4	40
Buysse et al., 1990	98	23	34.9	25.9
Buysse et al., 1990	41	23	34.3	26.3
Lauer et al., 1991	74	51	38.8	39
Mendlewicz & Kerkhofs, 1991	67	66	42	43.5
Lauer et al., 1992	14	12	38.6	54
Hudson et al., 1992	19	19	24.5	37
Moeller et al., 1993	34	36	47.5	100
Lauer et al., 1995	18	20	30.1	47.5
Hubain et al., 1995	15	20	38	100
Zarcone et al., 1995	11	10	34.8	100
Thase et al., 1996	90	12	38.2	43
Thase et al., 1996	20	12	37.7	43
Thase et al., 1997	44	22	32.6	41
Thase et al., 1997	181	22	36.3	36
Hoffman et al., 2000	13	13	32.2	100
Liscombe et al., 2002	10	10	30.5	0
Liscombe et al., 2002	10	10	30.5	100
Petaranderl et al., 2002	9	10	36.3	100
Staner et al., 2003	21	21	45.4	52
Motivala et al., 2005	22	18	42.4	100
Landolt & Gillin, 2005	16	16	41.2	50
Held et al., 2006	10	10	52.8	40
Hubain et al., 2006	67	67	42.7	100
Brower et al., 2011	10	10	29	100
Plante et al., 2012	19	19	23.3	0
Plante et al., 2012	11	11	30	100
Frey et al., 2012	9	8	25.8	0
Asaad et al. 2016	20	20	37.4	65
Goldschmied et al., 2018	37	59	28.5	54.8
Kwon et al., 2019	30	30	50.3	50
